# Спонтанная ремиссия нейроэндокринных заболеваний вследствие апоплексии в гормонально активные аденомы гипофиза

**DOI:** 10.14341/probl13567

**Published:** 2025-09-14

**Authors:** В. О. Вишнякова, Д. А. Беляева, Е. А. Старостина, Е. Г. Пржиялковская

**Affiliations:** Национальный медицинский исследовательский центр эндокринологии имени акад. И.И. Дедова; I.I. Dedov Endocrinology research centre

**Keywords:** апоплексия, акромегалия, болезнь Иценко-Кушинга, гипопитуитаризм, apoplexy, acromegaly, Cushing’s disease, hypopituitarism

## Abstract

This article presents a literature review on the topic of remission of severe neuroendocrine diseases due to adenoma apoplexy. The discussion addresses possible mechanisms underlying apoplexy in pituitary adenomas.

Additionally, two clinical cases of spontaneous remission of acromegaly and Cushing's disease in patients hospitalized at Endocrinology research Centre for neurosurgical treatment are discussed. These cases highlight the need for thorough examination and retesting of patients with hormonally active tumors immediately prior to neurosurgical interventions.

Апоплексия в аденому гипофиза представляет собой редкий, но потенциально смертельный клинический синдром, поражающий от 2 до 12% пациентов с аденомами гипофиза, встречающийся преимущественно у пациентов с гормонально неактивными аденомами. Первый летальный случай кровоизлияния в аденому гипофиза описал P. Bailey в 1898 г. Второй случай такого явления был описан в 1905 г. Bleibtreu L. в его работе «EinFallvonAkromegalie», когда при посмертном вскрытии головного мозга у молодого пациента с акромегалией было обнаружено геморрагическое поражение гипофиза. Основным клиническим проявлением данного синдрома является внезапное возникновение интенсивной головной боли, сопровождаемой зрительными нарушениями [1]. Факторы, предрасполагающие к развитию апоплексии в аденому гипофиза, включают артериальную гипертензию, нейрохирургические вмешательства, применение антикоагулянтной терапии и черепно-мозговые травмы. Апоплексия также может возникнуть после проведения определенных диагностических тестов, таких как введение инсулина, тиреотропин-рилизинг-гормона, гонадотропин-рилизинг-гормона, соматорелина, кортикотропин-рилизинг-гормона. Период между проведением исследования и началом апоплексии обычно составляет всего несколько минут [2–5].

Нормальная васкуляризация гипофиза поддерживается гипофизарной портальной системой, состоящей из сети капилляров, которые проходят от гипоталамуса через длинные воротные вены и верхнюю гипофизарную артерию, достигая передней доли гипофиза, а также из нижней гипофизарной артерии, питающей заднюю долю гипофиза. Обе эти артерии берут свое начало от внутренней сонной артерии и имеют анастомозы. Однако у аденом гипофиза васкуляризация осуществляется преимущественно прямым артериальным кровоснабжением, а не через портальную систему, что создает условия для возможных нарушений в кровоснабжении и повышения риска развития апоплексии. Кроме того, кровеносные сосуды аденом гипофиза характеризуются неполным созреванием и повышенной хрупкостью сосудистых стенок. С увеличением размеров новообразования гипофиза растет потребность в кислороде, что также может привести к апоплексии [6–9].

Клиническая картина апоплексии гипофиза варьирует и в значительной степени определяется степенью кровоизлияния, некроза и отека. Обычно характеризуется внезапным началом сильной головной боли, которая отмечается более чем у 80% пациентов, сопровождаемой рвотой (57%), нарушениями зрения (50%) и симптомами паралича черепно-мозговых нервов (52%). Головная боль представляет собой доминирующий симптом острой апоплексии гипофиза, который отмечается более чем у 80% пациентов, и ее внезапное и интенсивное наступление часто описывается пациентами как «раскат грома среди ясного неба» [1][10][11]. Предположительно, такая симптоматика обусловлена тракцией твердой мозговой оболочки или проникновением крови и некротического материала в субарахноидальное пространство, что вызывает раздражение мозговых оболочек. В большинстве случаев головная боль имеет ретроорбитальный характер, однако может проявляться бифронтальной или диффузной болью. Часто головная боль сопровождается рвотой и тошнотой, что может маскироваться под симптомы мигрени или менингита. В редких случаях головная боль протекает в бессимптомной или подострой форме с постепенным нарастанием симптомов, и тогда апоплексия в аденому может быть случайно обнаружена при проведении инструментальных визуализирующих исследований или вскрытии [1][10][11]. Другими частыми симптомами апоплексии являются потеря зрения, диплопия и офтальмоплегия. Специфическим эндокринным осложнением является развитие гипопитуитаризма с потерей одной или нескольких функций гипофиза.

Диагностика осуществляется с использованием компьютерной томографии (КТ) или магнитно-резонансной томографии (МРТ) головного мозга. МРТ является предпочтительным методом диагностики апоплексии, так как обладает более высокой чувствительностью по сравнению с КТ — от 88 до 90%. Типичные признаки апоплексии на МРТ: в острой фазе (0–7 дней) обнаруживается гипоинтенсивный сигнал на Т2-взвешенной визуализации (T2W1) с изоинтенсивностью или незначительной гипоинтенсивностью на Т1-взвешенной визуализации (T1W1), в подострой фазе (7–21 день) кровоизлияние становится гиперинтенсивным как на Т1-взвешенной визуализации, так и на Т2-взвешенной визуализации, в хронической фазе (>21 дня) наблюдается выраженная гипоинтенсивность как на Т1-взвешенной визуализации, так и на Т2-взвешенной визуализации [12].

Апоплексия в аденому гипофиза ранее рассматривалась как неотложное нейрохирургическое состояние. В настоящее время наиболее часто прибегают к консервативной терапии. Результаты исследований подтверждают позицию, что выжидательная тактика приводит к самостоятельному разрешению зрительных нарушений и восстановлению функции гипофиза [13].

После рассмотрения общих аспектов диагностики и лечения апоплексии в аденомы гипофиза мы хотим представить конкретные клинические случаи, демонстрирующие возможные исходы данного состояния. Особый интерес представляют редкие случаи спонтанной ремиссии тяжелых нейроэндокринных заболеваний, таких как акромегалия и болезнь Иценко-Кушинга вследствие апоплексии в аденомы гипофиза.

## МЕТОДЫ

Представленная статья содержит истории болезни наших пациентов, анализ соответствующей литературы в PubMed, eLibrary, CYBERLENINCA и данные пациентов из медицинской информационной системы qMS ГНЦ ФГБУ «НМИЦ эндокринологии» Минздрава Российской Федерации (МЗ РФ).

Нами были проанализированы ранее опубликованные клинические случаи ремиссии нейроэндокринных заболеваний после апоплексии в аденому гипофиза. Для этого был проведен поиск в базе данных PubMed, eLibrary и CYBERLENINCA с 2010 по 2024 гг. с использованием ключевых слов «апоплексия» и «ремиссия».

Критерии включения: подтвержденный диагноз гормонально-активной аденомы гипофиза, визуализационные данные апоплексии в аденому гипофиза, доказанная ремиссия нейроэндокринного заболевания после эпизода апоплексии, отсутствие ограничений по возрасту и полу, наличие информированного согласия от пациентов и их законных представителей на использование медицинских данных в исследовательских целях.

Критерии исключения: отсутствие подтвержденной аденомы, другие причины развившегося состояния, отсутствие полных данных по гормональному статусу или клиническому состоянию пациента. Первым этапом мы просмотрели заголовки и аннотации, чтобы определить, соответствуют ли статьи критериям включения, вторым — оценили содержание статей на соответствие нашим критериям включения. Мы разработали стандартизированную форму для извлечения данных, которая включала: идентификационные данные статьи (авторы, год публикации, журнал), характеристики пациента (возраст, пол), тип нейроэндокринного заболевания до апоплексии (диагностические критерии), характеристики аденомы гипофиза (размер), клинические проявления апоплексии, лечение апоплексии (консервативное, хирургическое), сопутствующую дисфункцию других желез внутренней секреции или нарушение продукции других гормонов гипофиза после апоплексии, наличие ремиссии основного заболевания. Проанализировав данные, мы систематизировали результаты в виде таблицы, включающей в себя: длительность заболевания до апоплексии в аденому гипофиза, размеры аденомы гипофиза, пол и возраст пациентов, клинические проявления, возможные триггеры апоплексии, исход основного заболевания и осложнения, а также лечение, которое проводилось данным больным.

Кроме того, с помощью медицинской информационной системы qMS ГНЦ ФГБУ «НМИЦ эндокринологии» МЗ РФ мы произвели выгрузку историй болезней всех пациентов нашего центра с диагнозом «Апоплексия в аденому гипофиза» с октября 2015 по июнь 2024 г. и также провели анализ клинических данных о ремиссии нейроэндокринных заболеваний вследствие апоплексии в аденому гипофиза.

## Спонтанная ремиссия акромегалии вследствие апоплексии в аденому гипофиза

Пациентка, 46 лет, с акромегалией поступила в отделение нейроэндокринологии ФГБУ «НМИЦ эндокринологии» Минздрава России в ноябре 2023 г. с жалобами на слабость, утомляемость, головные боли и боли в суставах.

Из анамнеза

Впервые отметила появление симптомов акромегалии в 2021 г.: укрупнение черт лица и увеличение размера обуви. За медицинской помощью не обращалась до августа 2023 г., когда впервые была госпитализирована на стационарное лечение по месту жительства по поводу головной боли, не купирующейся приемом нестероидных противовоспалительных средств (НПВС), повышением артериального давления (АД) до 170/100 мм рт.ст. На МРТ головного мозга с контрастным усилением, выполненной 31 августа 2023 г., визуализировано объемное образование хиазмально-селлярной области с четкими контурами, размерами 2,5х2,2х2,6 см, с тесным прилежанием к сифонам внутренних сонных артерий, с супра- интраселлярным распространением и компрессией хиазмы. По данным лабораторного обследования подтверждена активная стадия акромегалии: повышение уровня инсулиноподобного фактора роста 1 (ИФР-1) до 663 мкг/л (норма до 218). Пациентка была дистанционно консультирована врачом-эндокринологом ФГБУ «НМИЦ эндокринологии» Минздрава России, рекомендовано нейрохирургическое лечение в плановом порядке.

В ноябре 2023 г. впервые госпитализирована в отделение нейроэндокринологии ФГБУ «НМИЦ эндокринологии» Минздрава России для обследования на предмет осложнений акромегалии и подготовки к нейрохирургическому лечению. При поступлении в отделение ведущими жалобами пациентки были выраженная слабость, головные боли и эпизоды снижения АД, отсутствие менструаций с марта 2023 г. В ходе обследования подтверждена ремиссия акромегалии: уровень ИФР-1 в пределах нормальных значений — 120,6 нг/мл (15–250), подавление соматотропного гормона (СТГ) в ходе перорального глюкозотолерантного теста максимально до 0,132 нг/мл (норма менее 0,4 нг/мл). Также впервые были выявлены вторичная надпочечниковая недостаточность, вторичный гипотиреоз и гипогонадотропный гипогонадизм (табл. 1).

**Table:** Таблица 1. Гормональные исследования крови в ФГБУ «НМИЦ эндокринологии» МЗ РФ

Кортизол утром	↓86,97	нмоль/л	64–327
свТ4	↓9,7	пмоль/л	15–19
Эстрадиол	↓46,72	пмоль/л	97–592
ИПФР-1	N 120,6	нг/мл	15–250
СТГ в ходе ПГТТ	N 0,132	нг/мл	менее 0,4 нг/мл
ФСГ	3,3	Ед/л	1,6–9,7
ЛГ	↓0,873	Ед/л	2,5–11

На МРТ головного мозга с контрастным усилением — МР-признаки перенесенной апоплексии в аденому гипофиза: в полости турецкого седла с распространением в левый кавернозный синус визуализируется образование неправильной формы, неоднородного МР-сигнала за счет кистозного компонента в центральных отделах с гиперинтенсивным на Т1-ВИ ободком по контуру. При сравнении со снимками МРТ от 31.08.2023 отмечается уменьшение размеров макроаденомы гипофиза с 25х22х26 мм до 26х13х22 мм (рис. 1).

**Figure:**
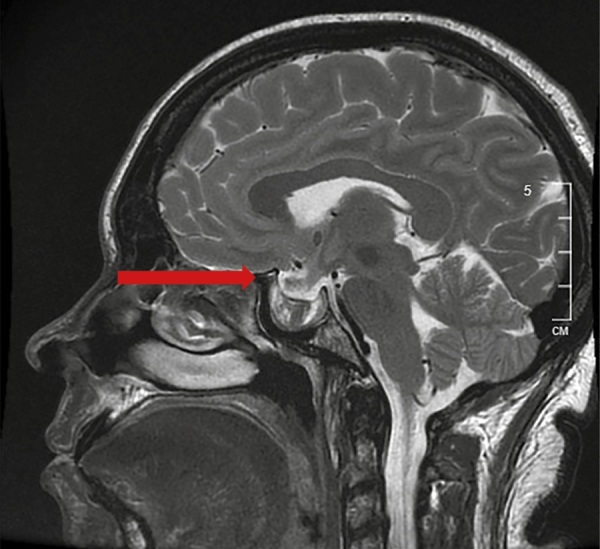
Рисунок 1. МРТ головного мозга с контрастным усилением от 23.11.2023. МР-признаки перенесенной апоплексии в аденому гипофиза.

Таким образом, подтверждена ремиссия акромегалии вследствие апоплексии в аденому гипофиза, нейрохирургическое лечение не показано, установлен гипопитуитаризм. Инициирована терапия гидрокортизоном в дозировке 10 мг в 08:00 и 5 мг в 16:00. Через неделю к терапии добавлен левотироксин натрия 50 мкг утром. На фоне проводимой терапии пациентка отметила значительное улучшение самочувствия.

В заключение данного случая следует отметить, что, несмотря на ремиссию акромегалии, апоплексия в аденому гипофиза осложнилась развитием гипопитуитаризма, что потребовало дополнительного назначения гормональной терапии и дальнейшего наблюдения за пациенткой. Этот случай подчеркивает необходимость тщательного мониторинга пациентов после апоплексии для своевременной коррекции возможных осложнений.

## Спонтанная ремиссия болезни Иценко-Кушинга вследствие апоплексии в аденому гипофиза

Пациент, 45 лет, с болезнью Иценко-Кушинга, госпитализирован в отделение нейроэндокринологии ФГБУ «НМИЦ Эндокринологии» МЗ РФ в июне 2020 г.

Из анамнеза

В марте 2020 г. обратился к эндокринологу по месту жительства с жалобами на быстрое увеличение массы тела на 15 кг в течение 1,5 года, боли в мышцах нижних конечностей, повышение АД до 160/100 мм рт.ст., сопровождаемое головной болью, появление багровых стрий на боковых поверхностях живота. В ходе лабораторно-инструментального обследования подтверждена болезнь Иценко-Кушинга: повышение уровня кортизола суточной мочи до 526,9 нмоль/л (норма — менее 485), отрицательный ночной подавляющий тест с 1 мг дексаметазона: кортизол утром — 154 нмоль/л (норма менее 50), адренокортикотропный гормон (АКТГ) — 95 пг/мл. На МРТ головного мозга с контрастным усилением выявлено эндоселлярное образование гипофиза неправильной округлой формы, размерами 9х8,5х10,6 мм.

В июне 2020 г. впервые госпитализирован в отделение нейроэндокринологии ФГБУ «НМИЦ эндокринологии» Минздрава России для обследования на предмет гиперкортицизма и подготовки к трансназальной транссфеноидальной аденомэктомии. Однако при обследовании получены низкие уровни вечернего кортизола крови и кортизола суточной мочи, что исключает наличие гиперкортицизма (кортизол суточной мочи — 78,4 нмоль/сут (100–379), вечерний кортизол слюны — 1,9 нмоль/л (0,5–9,65)). Данные лабораторных исследований представлены в таблице 2. На МРТ гипофиза с контрастным усилением визуализирована микроаденома гипофиза с признаками перенесенного кровоизлияния: в левой части аденогипофиза определялась зона кистозных изменений, преимущественно гиперинтенсивная с гипоинтенсивным ободком на Т2-взвешенных изображениях, размерами 5х8 мм, при контрастном усилении характеризующаяся сниженным (по сравнению с тканью аденогипофиза) накоплением контрастного препарата.

**Table:** Таблица 2. Гормональные исследования крови в ФГБУ «НМИЦ эндокринологии» МЗ РФ, подтверждающие ремиссию заболевания

Кортизол вечером	N 95,9	нмоль/л	64–327
Кортизол в ходе НПТ	N 11,6	нмоль/л	менее 50
АКТГ утром	N 26,8	пг/мл	7,2–63,3
АКТГ вечером	N 18,7	пг/мл	2–25,2
Кортизол суточной мочи	↓78	нмоль/сут	100–379
Кортизол в слюне вечером	N 1,93	нмоль/л	0,5–9,65

С целью обследования на предмет возможных осложнений апоплексии в аденому гипофиза исследован уровень кортизола крови утром, тиреотропного гормона, свободного тироксина, тестостерона, инулиноподобного фактора роста 1, пролактина. Выявлена вторичная надпочечниковая недостаточность: утренний кортизол крови — 48,85 нмоль/л (норма от 171 нмоль/л). Данных за нарушения других функций гипофиза не получено: ТТГ — 1,2 мМЕ/л (норма 0,25–3,5), свТ4 — 10,68 пмоль/л (норма 9–19), ИФР-1 224,7 нг/мл (норма 62,0–230,0), тестостерон 13,8 нмоль/л (норма 11,00–28,20), пролактин — 264 мЕд/л (норма 94–500).

Следовательно, в ходе стационарного обследования в отделении нейроэндокринологии подтверждена ремиссия болезни Иценко-Кушинга вследствие апоплексии в аденому гипофиза с развитием желательного исхода лечения — вторичной надпочечниковой недостаточности без нарушения других функций гипофиза. Нейрохирургическое лечение не показано, инициирована терапия гидрокортизоном 15 мг в 08:00 и 5 мг в 16:00, на фоне которой пациент отметил значительное улучшение общего самочувствия.

## ОБСУЖДЕНИЕ

Мы проанализировали ранее опубликованные клинические случаи ремиссии нейроэндокринных заболеваний после апоплексии в аденому гипофиза. Для этого был проведен поиск в базе данных PubMed, eLibrary, CYBERLENINCA с 2010 по 2024 гг. с использованием ключевых слов «апоплексия» и «ремиссия». В результате поиска выявлено 702 статьи, из которых 19 (n=19) соответствовали критериям отбора. Все ранее зарегистрированные случаи, а также наши два текущих случая, суммированы в таблице 3.

**Table:** Таблица 3. Обзор литературы о случаях ремиссии нейроэндокринных заболеваний после апоплексии в аденому гипофиза

№	Публикация	Диагноз	Длительность заболевания	Размеры аденомы гипофиза, см	Пол, возраст	Симптомы	Триггер	Гипопитуитаризм	Лечение
1	Клинический случай №1	Акромегалия	~2 года	2,5х2,2х2,6	Ж, 46	-	-	Вторичная надпочечниковая недостаточность; вторичный гипотиреоз; гипогонадотропный гипогонадизм	Гидрокортизон, левотироксин натрия
2	Клинический случай №2	Болезнь Иценко-Кушинга	~1,5 года	0,9х0,85х0,106	М, 45	-	-		Гидрокортизон
3	[14]	Болезнь Иценко-Кушинга	~3 года	2,0х2,8х1,5	М, 33	Внезапное появление сильной головной боли и тошноты, рвоты, гипотония, битемпоральная гемианопсия и диплопия	-	Вторичный гипотиреоз; вторичный гипогонадизм	Экстренная транссфеноидальная операция и декомпрессия
4	[15]	Акромегалия	-	1,6х1,8	Ж, 51	Сильная головная боль, фонофобия, светобоязнь, тошнота и рвота, симптомы Кернига и Брудзинского, температура 38 ºC	-	Вторичная надпочечниковая недостаточность; вторичный гипотиреоз, несахарный диабет, вторичный гипогонадизм	-
5	[16]	Акромегалия	~ 5-10 лет	-	М, 41	Сильная боль в шее	-	Вторичная надпочечниковая недостаточность; вторичный гипогонадизм	Гидрокортизон и препараты тестостерона
6	[16]	Болезнь Иценко-Кушинга	~1 год	-	Ж, 47	Острая головная боль	-	Последующий рецидив болезни Иценко-Кушинга	Гидрокортизон
7	[17]	Болезнь Иценко-Кушинга	~17 лет	0,9х0,6х0,8	Ж, 15		-		Последующий рецидив болезни Иценко-Кушинга с ремиссией после транссфеноидальной аденомэктомией
8	[18]	Акромегалия	~1 год	3,2х3,0х3,2	М, 18	Сильная головная боль, периодическая рвота и нечеткость зрения, битемпоральная гемианопсия	-	-	-
9	[19]	Акрогигантизм	-	0,7 -> 0,3 в высоту	Ж, 11	Умеренные головные боли без нарушений зрения, с рвотой, лихорадкой, вялостью и потерей веса на 6 кг за 3 недели	-	СТГ-дефицит; вторичный гипогонадизм; вторичный гипотиреоз	Гормон роста, левотироксин натрия, эстрадиол
10	[20]	Акромегалия	~10 лет	0,12	М, 40	Интенсивная головная боль в течение 4–5 часов	-	-	-
11	[21]	Болезнь Иценко-Кушинга	~ 3 года	-	Ж, 31	Головокружение, тошнота, рвота, потеря аппетита, интенсивная головная боль	-	Гипогонадотропный гипогонадизм; вторичный гипотиреоз	Гидрокортизон
12	[22]	Болезнь Иценко-Кушинга	~2 года	1,5х1,9х2,3	Ж,59	Острая головная боль, тошнота	-	-	-
13	[23]	Акромегалия	~5 лет	-	М, 50	Острая головная боль	-	Пангипопитуитаризм с последующим восстановлением	Гидрокортизон
14	[24]	Болезнь Иценко-Кушинга	-	0,1х0,15х0,18	Ж, 77	Острая головная боль, тошнота, рвота	-	Пангипопитуитаризм	Гидрокортизон
15	[25]	Болезнь Иценко-Кушинга	-	правая аденома — 0,4х0,3 левая аденома — 0,8х0,6 (в нее апоплексия)	М, 18	Постоянная рвота на фоне надпочечниковой недостаточности	Ветряная оспа	Вторичная надпочечниковая недостаточность	Гидрокортизон
16	[26]	Акромегалия	-	0,26х0,21х0,15	М, 36	Острая головная боль	-	Вторичная надпочечниковая недостаточность; вторичный гипотиреоз	Гидрокортизон, левотироксин натрия
17	[27]	Акромегалия	-	-	М, 32	Головная боль, тошнота, рвота	-	Вторичная надпочечниковая недостаточность; вторичный гипогонадизм; вторичный гипотиреоз; гипопролактинемия	Внутривенное введение глюкокортикостероидов (препарат не указан), проведение транссфеноидальной аденомэктомии в связи с компрессией зрительного перекреста
18	[28]	Акромегалия	~1,5 года	0,22х0,25х 0,32	М, 25	Головная боль, резкое снижение остроты зрения в правом глазу, западение правого верхнего века, повышение температуры тела, сухость во рту, частое мочеиспускание, общая слабость, тошнота, рвота	-	-	Транссфеноидальное удаление инфралатеросупраселлярной аденомы гипофиза
19	[29]	Акромегалия, гиперпролактинемия	~10 лет	0,2х0,27х0,3	Ж, 47	-	Нейрохирургическое вмешательство	Вторичный гипотиреоз	Левотироксин натрия, каберголин, октреотид длительного действия

Возраст пациентов колебался от 11 до 77 лет (средний возраст — 38 лет). Наиболее часто данная патология описывалась у мужчин (в 10 случаях). Средняя продолжительность заболевания до апоплексии гипофиза составила 5 лет. Только в 4 случаях из 19 отсутствовала яркая клиническая картина, характерная для апоплексии, у одного пациента описывались нетипичные проявления апоплексии, такие как симптомы Брудзинского и Кернига с повышением температуры тела.

Из 19 случаев только у 2 пациентов есть связь с провоцирующим фактором — апоплексия в аденому гипофиза, индуцированная ветряной оспой, а также нейрохирургическое вмешательство.

Во всех случаях апоплексия привела к ремиссии основного заболевания, хотя позднее у одной пациентки случился рецидив болезни Иценко-Кушинга. 12 пациентам потребовалась медикаментозная терапия гипопитуитаризма, возникшего после перенесенной апоплексии в аденому гипофиза. Наиболее часто гипопитуитаризм встречался у пациентов с болезнью Иценко-Кушинга (42,1%). У пациентов с акромегалией — в 57,9% случаев. В 10,5% случаев подтверждено развитие пангипопитуитаризма, у одного из которых в дальнейшем произошло восстановление всех функций гипофиза.

Частота развития осложнений распределилась следующим образом: у 57,9% пациентов диагностированы вторичный гипотиреоз и гипогонадотропный гипогонадизм, у 42,1% — вторичная надпочечниковая недостаточность, у 15,8% — СТГ-дефицит, у 5,3% — центральный несахарный диабет и у 5,3% — гипопролактинемия.

За время работы ГНЦ ФГБУ «НМИЦ эндокринологии» МЗ РФ с октября 2015 по июнь 2024 гг. зарегистрировано 9 случаев апоплексии в гормонально активную аденому гипофиза (n=9). Из них только у 2, ранее описываемых, пациентов развилась ремиссия основного заболевания, у 4 пациентов наблюдалось развитие гипопитуитаризма: у 4 (44%) — вторичная надпочечниковая недостаточность, у 3 (33%) — вторичный гипотиреоз, у 2 (22%) — гипогонадотропный гипогонадизм, у 2 (22%) — центральный несахарный диабет.

## ЗАКЛЮЧЕНИЕ

Апоплексия гипофиза может возникнуть у пациентов с аденомами гипофиза, и ее следует предполагать у любого пациента, у которого появилась острая сильная головная боль. Помимо историй болезней пациентов нашего центра, мы проанализировали данные зарубежной литературы и обнаружили, что в 71% случаев головная боль была ведущим симптомом. Однако у части пациентов клинические проявления отсутствовали, поэтому важно помнить о неспецифических симптомах кровоизлияния в аденому гипофиза (тошнота, рвота, нарушение зрения, головокружение, лихорадка), а также о проявлениях снижения выработки гормонов гипофиза, например, внезапное прекращение менструального цикла у женщин, снижение потенции у мужчин, гипотония, полиурия, полидипсия.

После апоплексии необходима повторная оценка секреции гормонов гипофиза с целью решения вопроса о необходимости инициации соответствующей терапии. У пациентов с гормонально активными аденомами важно проводить ретестирование перед нейрохирургическими вмешательствами, что позволит определить актуальный статус активности заболевания и принять обоснованное решение о необходимости оперативного лечения.

Спонтанная ремиссия тяжелых нейроэндокринных заболеваний, вызванная апоплексией в аденому гипофиза, может казаться положительным исходом. Однако важно подчеркнуть, что такие события могут сопровождаться серьезными осложнениями, включая нарушения зрения и развитие гипопитуитаризма, что диктует необходимость тщательного мониторинга пациентов после апоплексии.

## ДОПОЛНИТЕЛЬНАЯ ИНФОРМАЦИЯ

Источники финансирования. Работа выполнена по инициативе авторов без привлечения финансирования.

Конфликт интересов. Авторы декларируют отсутствие явных и потенциальных конфликтов интересов, связанных с содержанием настоящей статьи.

Участие авторов. Вишнякова В.О., Беляева Д.А., Старостина Е.А. — концепция и дизайн рукописи, написание рукописи; Пржиялковская Е.Г. — концепция и дизайн рукописи, внесение в рукопись существенных правок с целью повышения научной ценности статьи. Все авторы одобрили финальную версию статьи перед публикацией, выразили согласие нести ответственность за все аспекты работы, подразумевающую надлежащее изучение и решение вопросов, связанных с точностью или добросовестностью любой части работы.

Благодарности. Авторы выражают глубокую признательность врачам-эндокринологам, которые своевременно диагностировали акромегалию и болезнь Иценко-Кушинга и направили пациентов в ФГБУ «НМИЦ эндокринологии» Минздрава России для специализированного лечения. Также мы благодарим пациентов, согласившихся на использование их клинических данных и историю болезни для описания в данной публикации, что позволило внести важный вклад в развитие научного знания и практики в области эндокринологии.
